# Bridging Gaps and Charting Future Directions in Vasculitis

**DOI:** 10.3390/jcm13216571

**Published:** 2024-11-01

**Authors:** Ryu Watanabe

**Affiliations:** Department of Clinical Immunology, Osaka Metropolitan University Graduate School of Medicine, Osaka 545-8585, Japan; doctorwatanaberyu7@gmail.com; Tel.: +81-6-6645-3981

The field of vasculitis continues to evolve rapidly, driven by breakthroughs in both basic and clinical research [1]. Despite the strides made, unmet clinical needs remain in the diagnosis and treatment of vasculitis, such as delayed diagnosis, heterogeneous disease presentations, limited biomarkers, and treatment side effects [2,3,4]. Recognizing the urgency of addressing these issues, this Special Issue of the Journal of Clinical Medicine was designed to provide a platform for innovative research, with the goal of advancing knowledge in these areas.

The six articles included in this Special Issue span a diverse range of topics, such as systemic necrotizing vasculitides (SNVs) and antineutrophil cytoplasmic antibody (ANCA)-associated vasculitis, each offering unique perspectives to existing knowledge. These contributions not only enhance our understanding of vasculitis but also open new avenues for clinical applications and translational research.

The articles published herein exemplify the central objective of this Special Issue, which was to bridge existing knowledge gaps and foster interdisciplinary collaboration, by addressing key challenges. For example, Shumnalieva R, et al. demonstrated that calprotectin, LAMP-2 antibody, and matrix metalloproteinase-9 could be novel biomarkers for evaluating disease activity [5]. In addition, Houben E, et al. showed that a wide variety of diseases can mimic ANCA-associated vasculitis, including not only other vasculitides and autoimmune diseases, but also infectious diseases such as infective endocarditis and mycobacterium, malignancies such as lung cancer and malignant lymphoma, and even cocaine abuse [6].

While the contributions in this Special Issue offer meaningful advancements, several areas demand further exploration. Future research should prioritize the following topics.

## 1. Giant Cell Arteritis (GCA)

### 1.1. Diagnosis

The utility of non-invasive imaging modalities such as ultrasound and positron emission tomography for early diagnosis is increasingly recognized [7,8], but further validation is needed.

### 1.2. Disease Activity Assessment

There is a lack of universally accepted biomarkers for assessing disease activity and predicting flares [9]. This makes it challenging to monitor treatment response and guide therapy decisions.

### 1.3. Treatment

While glucocorticoids (GCs) remain the mainstay of treatment, their long-term use is associated with significant side effects [10]. Although tocilizumab has shown efficacy in recent trials [11], its long-term efficacy and safety require further investigation. Recently, the efficacy of Janus kinase inhibitors (JAKi) has been suggested [12], but, alongside their safety, this requires verification in daily clinical practice. In particular, we need to pay close attention to the development of shingles.

## 2. Takayasu Arteritis (TA)

### 2.1. Diagnosis

TA is often diagnosed late due to its insidious onset and non-specific symptoms. There is a need for improved early diagnostic tools and increased awareness among clinicians.

### 2.2. Disease Activity Assessment

Similar to GCA, there is a lack of reliable biomarkers for assessing disease activity in TA [13]. The correlation between systemic inflammation and vascular wall inflammation is often poor, making it challenging to guide treatment decisions.

### 2.3. Treatment

While GCs are the initial treatment, many patients require additional immunosuppressive agents. The IL-6 receptor antibody tocilizumab has been shown to be effective [14], but relapses are still common. JAKi are also expected to be effective for TA [15], but the results of a large randomized controlled trial have not yet been published. In addition, the lack of suitable animal models for TA hinders research into disease mechanisms and the development of targeted therapies [16].

## 3. Polyarteritis Nodosa (PAN)

### 3.1. Diagnosis

The distinction between PAN and other forms of vasculitis, particularly ANCA-associated vasculitis, can be challenging. Improved classification or diagnostic criteria are needed.

### 3.2. Disease Activity Assessment

There is a lack of validated tools for assessing disease activity and predicting relapse in PAN.

### 3.3. Treatment

While GCs and cyclophosphamide are effective in many patients, refractory cases pose a significant challenge [17]. Due to the small number of cases [18], the pathogenesis of the disease remains unclear, and the role of biologic agents, such as rituximab, is not well established.

## 4. ANCA-Associated Vasculitis (AAV)

### 4.1. Diagnosis

Early diagnosis is crucial for preventing irreversible organ damage and improving patient outcomes. Thus, there is a need for improved biomarkers to facilitate earlier detection of AAV.

### 4.2. Disease Activity Assessment

Current methods, including ANCA titers, are not always reliable indicators of disease activity or impending flares. Better biomarkers are needed to accurately assess disease activity and predict relapses.

### 4.3. Treatment

Although in recent years, many treatment options have become available, such as rituximab and avacopan [19,20], there is a need for better stratification of patients to tailor treatment approaches based on individual disease characteristics, ANCA specificity, and genetic factors.

We also encourage researchers to adopt multi-center collaborations, enabling larger and more diverse patient cohorts to validate findings. Our group recently set up a multi-center vasculitis cohort and revealed that the anti-IL-5 antibody mepolizumab contributes not only to the control of disease activity but also to long-term survival in patients with eosinophilic granulomatosis with polyangiitis [21]. This is a typical example of a large cohort study revealing a new role of biologic agents. Additionally, the integration of emerging technologies, such as single-cell RNA sequencing and machine learning, will likely play a pivotal role in overcoming current limitations and enhancing the clinical relevance of future research [22].

In closing, we hope that this Special Issue serves as a catalyst for continued inquiry and collaboration in vasculitis. The collective insights presented within these pages not only contribute to the existing body of knowledge but also lay the groundwork for future innovations. We extend our gratitude to the contributing authors and reviewers, whose efforts have made this Special Issue a valuable resource for both researchers and clinicians.

We look forward to seeing the impact of these studies unfold and invite the scientific community to build upon these findings to address the remaining challenges in vasculitis. It is our hope that this Special Issue inspires further research and contributes meaningfully to improving patient outcomes and advancing the frontiers of medical science.
